# Supplementary material for: Transcriptomic and Metabolomic Research on the Germination Process of Panax ginseng Overwintering Buds
Source: Plants (Basel). 2024 Apr 8;13(7):1041. doi: 10.3390/plants13071041 (PMC11013764; doi:10.3390/plants13071041)
Supplement: Supplementary file 1 [file plants-13-01041-s001.zip › Table S1. List of qRT-PCR primers used in the experiment.pdf]

Table S1 Primers used in the experiment

Gene ID	Forward primer (5'→3')	Reverse primer (5'→3')
EVM0001137	CGTACAACTGGTATCGTGCTG	CAGCAGTGGTGGTGAACAT
EVM0002701	ATGAAGGAAAGATTGGTTGG	AAACTTGCTCTTAGTGACAG
EVM0003588	AACCCTATTTCTCGGATCAGGT	TCAGCAAATGGATCGAAAGC
EVM0004731	ACCGAAGTCTCTGATAGTGTAA	TAATCTCAGTGTAACCGGTG
EVM0005219	CTGTCACTAAGAGCAAGTTTGA	GCCCTTTCCGACATCTCCGTAA
EVM0007067	GACGATGATTGCTTTGTTGC	GGACGAAATCTGTACCATCT
EVM0007078	ATACTGTTGTTGAGCCCT	GAGATCACCAAAGCTAGG
EVM0009275	AAGGGCAAAGGTTCCAAG	TACCCTTACAGTCATTGC
EVM0014402	TCACCGTCAAGGATGCTAGA	CTGTGCCAACTCTGTGTAGT
EVM0020372	AATGGTGAAGGCTGGTTTTG	TCTCTTCGATTGGGCTTCAT
EVM0021434	AAGCCAAGGGTCAGCATC	GAAGACAGGGAGGACATGAT
EVM0022327	GCGAAGCCAGCCAATTCAAC	GTCGCCTATGGTGTTGTAAA
PgGAPDH	CACGGTCCCTGGAAGCA	CATTGACACCCACAACAAACAT
