# Supplementary figures and images for: Transcriptomic and Metabolomic Research on the Germination Process of Panax ginseng Overwintering Buds
Source: Plants (Basel). 2024 Apr 8;13(7):1041. doi: 10.3390/plants13071041 (PMC11013764; doi:10.3390/plants13071041)

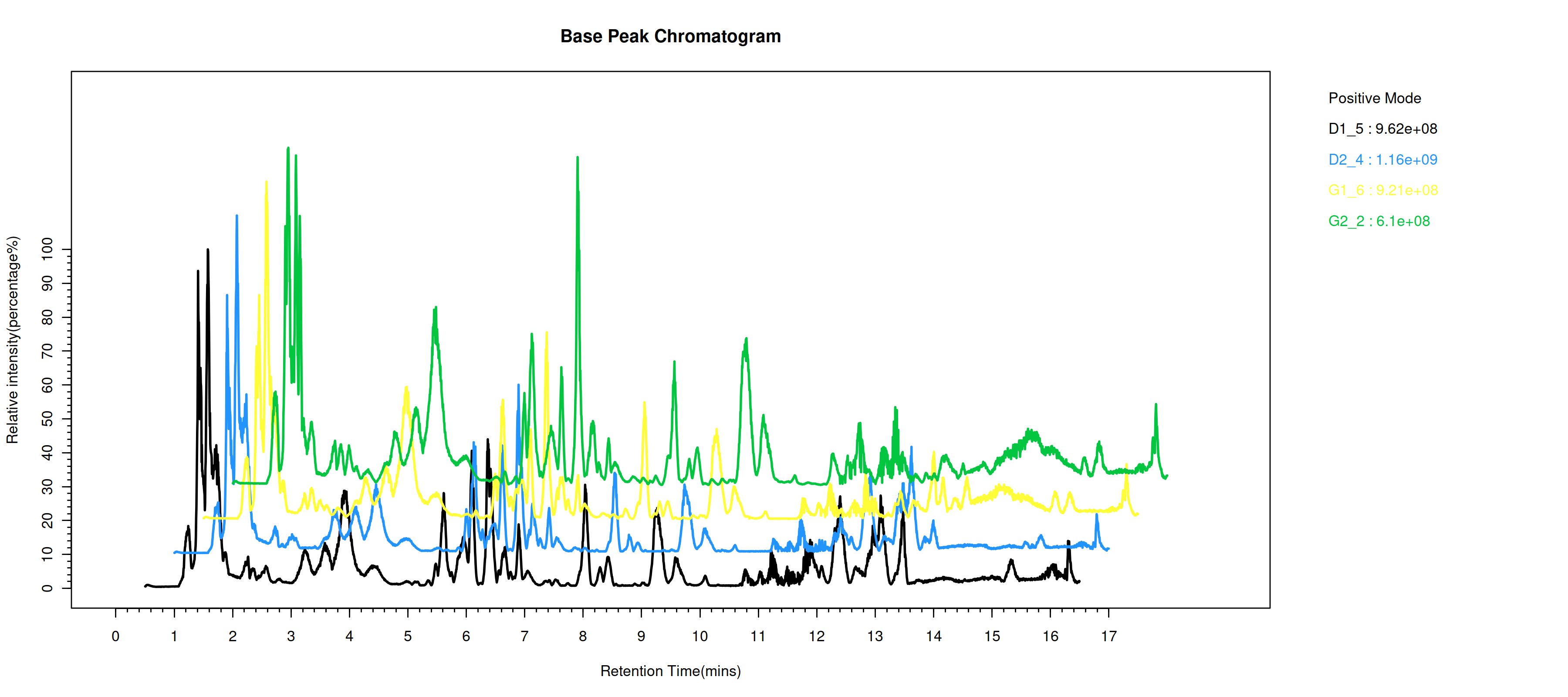

Supplement: Supplementary file 1 [file plants-13-01041-s001.zip › Fig.S1 Base peak chromatograms of four groups of samples.png]

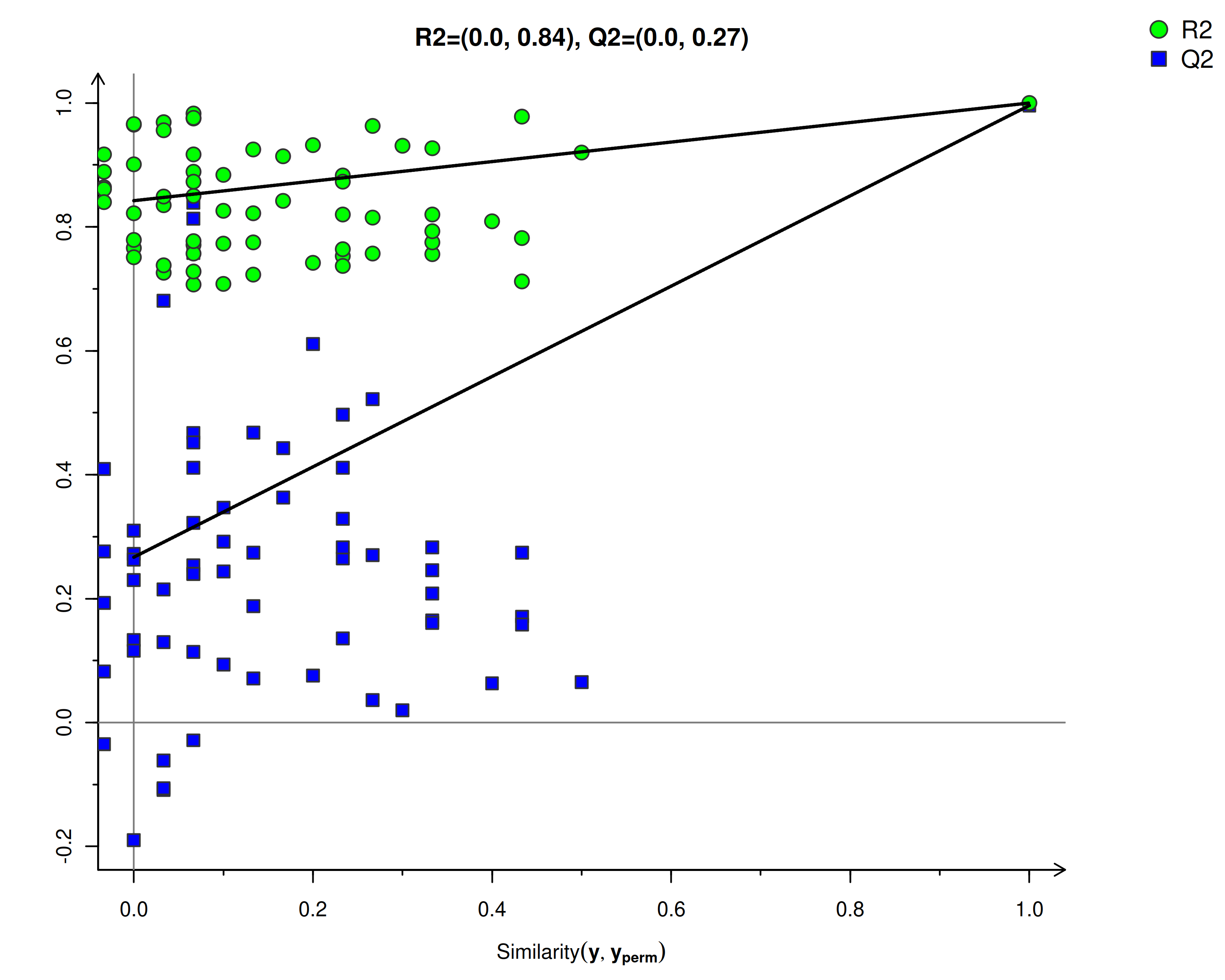

Supplement: Supplementary file 1 [file plants-13-01041-s001.zip › Fig.S2 PLS-DA permutation test plot of total metabolites.png]

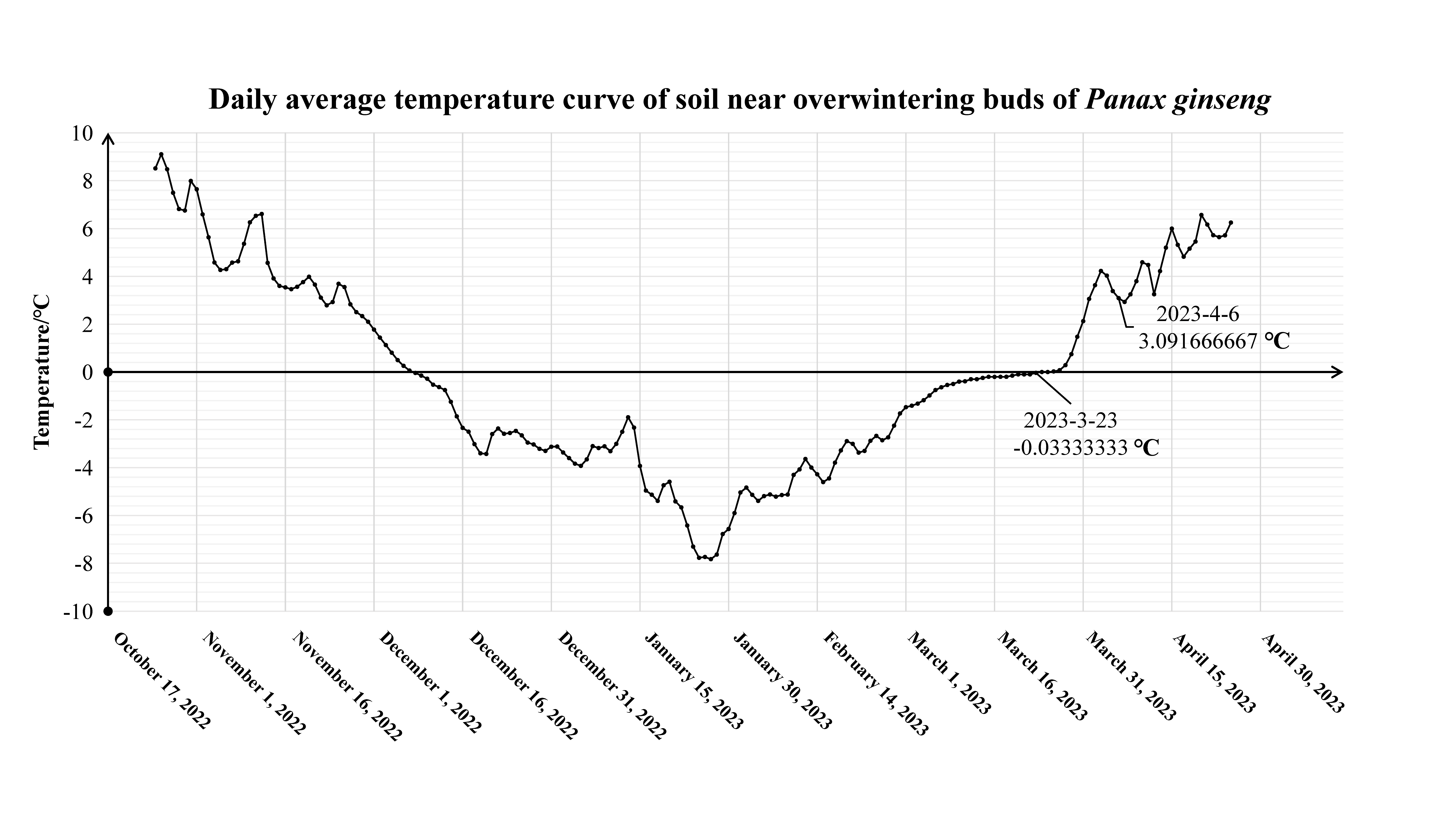

Supplement: Supplementary file 1 [file plants-13-01041-s001.zip › Fig.S3 Daily average temperature curve of soil near overwintering buds of Panax ginseng.png]
